# Supplementary material for: Violence and Clinical Learning Environments in Medical Residencies
Source: Int J Environ Res Public Health. 2023 Sep 13;20(18):6754. doi: 10.3390/ijerph20186754 (PMC10531318; doi:10.3390/ijerph20186754)
Supplement: Supplementary file 1 [file ijerph-20-06754-s001.zip › ijerph-2511259-supplementary.pdf]

International Journal of
Environmental Research and Public Health

MDPI

Supplementary materials:

Supplementary File 1. Section 3 RMLV questionnaire: Environment at the Clinical Site

3.1. Describe the environment of the site where you work as a resident based on the following adjectives.

**Very unpleasant**	**Unpleasant**	**Neither pleasant nor unpleasant**	**Pleasant**	**Very pleasant**
1	2	3	4	5
Very competitive	Competitive	Neither competitive nor cooperative	Cooperative	Very cooperative
1	2	3	4	5
Very tense	Tense	Neither tense nor relaxed	Relaxed	Very relaxed
1	2	3	4	5
Very conflictive	Conflictive	Neither conflictive nor constructive	Constructive	Very constructive
1	2	3	4	5

3.2. What would be the most typical reaction in your direct and daily environment on site (peers, colleagues) to a discriminatory comment? (Mark one option only)

A. Most would disapprove
B. Most would tolerate it
C. Most would approve and continue with the comment

3.3. In a hypothetical situation of abuse, do you think that:

Victims would run some type of risk if they reported it.	YES	NO
Aggressors would be penalized at the hospital if they were reported.	YES	NO
Supporting victims can lead to reprisals against those supporting the abused person.	YES	NO
The tolerance of abuse in the institution discourages people from reporting it.	YES	NO
People can be intimidated because abuse is normal at the hospital.	YES	NO

3.4. In your opinion, indicate how much you agree or disagree with the following statements.

1. Strongly disagree
2. Somewhat disagree
3. Neither agree nor disagree
4. Somewhat agree
5. Strongly agree

Punitive rotations are to maintain order among the residents. ~~in the department.~~
Reprimands are to prevent residents from committing errors.
Questioning by professors in front of everyone is to expose those who don't have the answer.
Tasks assigned to residents that are not academic or educational are best done to avoid conflict.
Profanity and vulgar expressions exclude women from the dynamic among residents.
Negative criticism from some residents toward others can lead to the resignation of the person criticized.
The strict application of hospital rules helps encourage a better academic and workplace environment.

Source: by the authors

Supplementary File 2. Perception of risk in hypothetical situations of violence in hospitals by number of residents and specialty

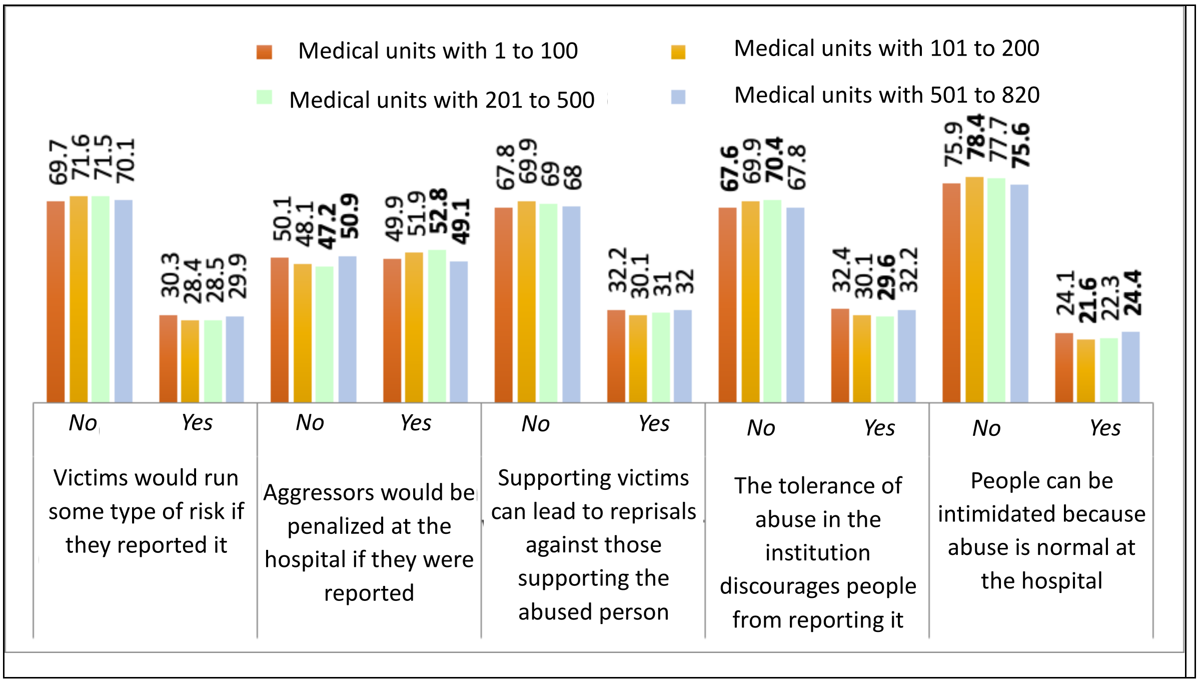

Note. Significant differences (p< .05) were obtained with the two-sided equality test for column proportions. Bonferroni correction was used. Differences are shown with percentages highlight-ed in bold Source: by the authors

Supplementary File 3. Opinions about common expressions of violence by year of residency (Prompt 3.4)

		Year of Residency										
		1st		2nd		3rd		4th		5th		
		n	%	*n*	%	*n*	%	*n*	%	*n*	%	X^2 (*p*)
Punitive rotations are to maintain order among the residents	Strongly disagree	4252	82.6	2821	77.0	1817	72.2	813	66.0	17	**53.1**	269.97 (0.001)
	Somewhat disagree	468	**9.1**	383	10.5	293	11.6	167	13.6	4	12.5	
	Neither agree nor disagree	247	**4.8**	269	7.3	224	8.9	137	11.1	4	12.5	
	Somewhat agree	127	**2.5**	137	3.7	139	5.5	81	6.6	7	21.9	
	Strongly agree	54	**1.0**	54	1.5	44	1.7	34	2.8	0[1]	0.0	
Reprimands are to prevent residents from committing errors	Strongly disagree	1925	37.4	1278	34.9	840	33.4	373	30.3	4	**12.5**	81.97 (0.001)
	Somewhat disagree	1254	24.4	894	24.4	561	22.3	266	21.6	6	18.8	
	Neither agree nor disagree	700	13.6	540	14.7	375	14.9	213	17.3	5	15.6	
	Somewhat agree	993	19.3	746	20.4	546	21.7	277	22.5	13	**40.6**	
	Strongly agree	276	5.4	206	5.6	195	7.7	103	8.4	4	12.5	
Questioning by professors in front of everyone is to expose those who don't have the answer	Strongly disagree	2375	46.1	1693	46.2	1138	45.2	551	44.7	11	34.4	24.50 (0.079)
	Somewhat disagree	835	16.2	605	16.5	470	18.7	215	17.5	9	28.1	
	Neither agree nor disagree	871	16.9	605	16.5	429	17.0	232	18.8	7	21.9	
	Somewhat agree	678	13.2	483	13.2	295	11.7	133	10.8	5	15.6	
	Strongly agree	389	7.6	278	7.6	185	7.4	101	8.2	0[1]	0.0	
Tasks assigned to residents that are not academic or educational are best done to avoid conflict.	Strongly disagree	2591	50.3	1810	49.4	1265	50.3	601	48.8	14	**43.8**	28.85 (0.025)
	Somewhat disagree	823	16.0	588	16.0	395	15.7	192	15.6	4	12.5	
	Neither agree nor disagree	785	15.2	583	15.9	424	16.8	244	**19.8**	6	18.8	
	Somewhat agree	605	11.8	438	12.0	303	12.0	120	9.7	4	12.5	
	Strongly agree	344	6.7	245	6.7	130	5.2	75	6.1	4	12.5	
Profanity and vulgar expressions exclude women from the dynamic among residents	Strongly disagree	2788	54.2	1914	52.2	1341	53.3	627	50.9	19	59.4	20.35 (0.205)
	Somewhat disagree	600	11.7	447	12.2	330	13.1	150	12.2	3	9.4	
	Neither agree nor disagree	931	18.1	711	19.4	485	19.3	272	22.1	6	18.8	
	Somewhat agree	441	8.6	317	8.7	189	7.5	103	8.4	2	6.3	
	Strongly agree	388	7.5	275	7.5	172	6.8	80	6.5	2	6.3	
Negative criticism from some residents toward others can lead to the resignation of the person criticized	Strongly disagree	1499	29.1	1098	30.0	772	30.7	341	27.7	12	37.5	81.01 (0.001)
	Somewhat disagree	528	10.3	400	10.9	284	11.3	146	11.9	2	6.3	
	Neither agree nor disagree	660	**12.8**	588	16.0	417	16.6	240	19.5	4	**12.5**	
	Somewhat agree	1336	26.0	904	24.7	577	22.9	281	22.8	13	**40.6**	
	Strongly agree	1125	21.9	674	18.4	467	18.6	224	18.2	1	**3.1**	
The strict application of hospital rules helps encourage a better academic and workplace environment	Strongly disagree	1771	**34.4**	1230	33.6	822	32.7	362	29.4	5	15.6	32.83 (0.008)
	Somewhat disagree	1020	19.8	735	20.1	474	18.8	214	17.4	9	**28.1**	
	Neither agree nor disagree	1107	21.5	770	21.0	556	22.1	307	24.9	8	25.0	
	Somewhat agree	899	17.5	664	18.1	481	19.1	258	20.9	8	25.0	
	Strongly agree	351	6.8	265	7.2	184	7.3	91	7.4	2	6.3	

Note: [1] This category was not used in comparisons because its column proportion equals zero or one. Significant differences ($p < 0.05$) were obtained with the two-sided equality test for column proportions. Bonferroni correction was used. The tests assume equal variances. Source: By the authors.
